# Fragmentation of Long Reads Enables Reliable Mitogenome Assembly From Whole‐Genome Amplification Data With Pervasive Palindromic Reads

**DOI:** 10.1111/1755-0998.70165

**Published:** 2026-06-14

**Authors:** Matteo Vecchi, Bartłomiej Surmacz, Ingemar K. Jönsson, Daniel Stec

**Affiliations:** ^1^ Department of Chemistry, Life Sciences and Environmental Sustainability University of Parma Parma Italy; ^2^ Institute of Systematics and Evolution of Animals, Polish Academy of Sciences Kraków Poland; ^3^ Department of Environmental Science Kristianstad University Kristianstad Sweden

**Keywords:** Acutuncus, chimeric sequences, long reads, mitochondria, nanopore, tardigrades

## Abstract

Whole genome amplification (WGA), and in particular multiple displacement amplification (MDA), has become a key technique for genomic sequencing of microscopic organisms, yet it introduces artefacts such as palindromic (inverted chimeric) reads that may compromise downstream analyses. We assessed how pervasive palindromic reads generated by MDA impact the assembly of tardigrade (*Acutuncus giovanniniae* and *A. mecnuffi*) mitogenomes sequenced with Oxford Nanopore technology. We show that the MDA produces a high proportion of palindromic reads, often exceeding one‐third of mitochondrial reads and frequently exhibiting complex multi‐inversion structures. These artefacts severely impair long‐read assembly, leading to low success rates and inconsistent genome reconstruction. To solve this issue, a strategy based on in silico fragmentation of long reads into short, high‐quality fragments, followed by short‐read assembly, consistently produced complete and accurate circularised mitochondrial genomes. Our results demonstrate that palindromic read formation can be, in some cases, a limitation of MDA coupled with long‐read sequencing, but this issue can be mitigated through read fragmentation. This approach provides a simple, robust and scalable solution for mitogenome assembly from data heavily affected by amplification artefacts, particularly in microscopic taxa where whole genome amplification is often unavoidable.

## 1 Introduction

The use of mitochondrial genomes in resolving phylogenetic relationships among different animal taxa has undoubtedly brought progress to the field of phylogenetics and systematics (e.g., Forni et al. 2021; García‐Souto et al. 2025; Lavrov et al. 2023; Sun et al. 2020). While first restricted by the inherent limitations of Sanger sequencing (Zardoya and Suárez 2008), the advent of second (454, Illumina and IonTorrent sequencing) and third (PacBio and Oxford Nanopore) generation massive parallel sequencing technologies were the principal drivers behind the sequencing and release of a massive amount of new genomic data. Among the third‐generation sequencing technologies, the small and cheap Oxford Nanopore Technologies MinION sequencer allowed even small laboratories to obtain high‐quality genomic data (Menegon et al. 2017; Parker et al. 2017). Yet a major limitation of these sequencing technologies is the amount of high‐molecular‐weight input DNA required (Pollard et al. 2018). While, in principle, enough material for library preparation and sequencing could be obtained from a single fly (Kim et al. 2024), obtaining enough DNA for microscopic metazoans or single‐cellular organisms is usually not possible. Although pooling of individuals is a potential solution (Flot et al. 2013; Li et al. 2024), it can heavily reduce the assembled genome quality due to high heterozygosis and contaminations (Bemm et al. 2016; Huang et al. 2017; Simion et al. 2018).

Whole genome amplification (WGA) refers to a series of methodologies that enable non‐selective amplification of the whole genome sequence (Wang et al. 2022). In particular, multiple displacement amplification (MDA) relies on isothermal amplification with Phi29 DNA polymerase and random exonuclease‐resistant primers (Dean et al. 2002), enabling high yield and accuracy from a small amount of the starting DNA template, combined with a straightforward laboratory protocol (Wang et al. 2022). However, this methodology has known limitations, including high variance in amplification efficiency (high variance of coverage and sequencing depth) across genomic regions due to GC amplification biases and differential binding efficiency of primers (Benita 2003; Borgström et al. 2017; Lee et al. 2023). Another well‐recognised issue with MDA is the production of inverted chimeric sequences consisting of one or more inverted repeats (palindromes) when 3′ termini are displaced and annealed to nearby displaced 5′ strands (Lasken and Stockwell 2007; Lu et al. 2023; Sabina and Leamon 2015; Warris et al. 2018). Bioinformatic pipelines have been developed to find and correct palindromic reads (Agyabeng‐Dadzie et al. 2025; Lee et al. 2023; Lu et al. 2023; Warris et al. 2018). However, one of these methods relies on mapping the reads to a reference genome (Lu et al. 2023), which limits its utility to already well‐characterised organisms. Other methods only identify palindromes, which are then discarded completely (Agyabeng‐Dadzie et al. 2025). Some approaches aim to correct palindromic reads (Lee et al. 2023) by splitting them in half in each software run iteration. Importantly, this approach is limited in its efficacy to situations where reads experienced only one or two palindrome formation events.

The development and commercialisation of WGA kits was particularly welcomed in the field of meiofauna research. The small size of meiofauna impacts both the effort put into their research due to difficulties in finding and identifying them (Fontaneto et al. 2015; Mammola et al. 2023; Martínez et al. 2025) and the amount of DNA available for massive parallel sequencing, leading to their underrepresentation in genomic and mitogenomic sequencing projects and studies (Kocot et al. 2017; Laumer et al. 2019). It comes naturally that there is a rise in the adoption of WGA for the sequencing of meiofauna genomes (e.g., Kosakyan et al. 2026; Roberts et al. 2024; Vecchi and Stec 2025). Therefore, the commercial availability of MDA kits brought forward the sequencing of genomes and mitogenomes of meiofauna from single individuals, with both second‐ and third‐generation massive parallel sequencing technologies (Camarda et al. 2025; Gammuto et al. 2024; Kosakyan et al. 2026; Lee et al. 2023; Roberts et al. 2024; Vecchi and Stec 2025). However, an investigation into how palindromic reads affect mitogenome assembly has not been performed.

Given the possibility to scale up the sequencing of mitogenomes of meiofauna provided by MDA, we used tardigrades as a focal system because they are microscopic animals for which single‐individual mitogenome sequencing is often constrained by low DNA input and therefore depends on WGA‐based approaches. We investigate how MDA with a high reaction gain coupled with long‐read sequencing (Oxford Nanopore) is impacted by the formation of palindromic reads, and we propose an approach, based on splitting long reads into short fragments that mimic the output of short‐read sequencing technologies, to efficiently produce mitogenome assemblies from data heavily impacted by this issue.

## 2 Material and Methods

### 2.1 Samples

Live tardigrades belonging to the species *Acutuncus giovanniniae* Vecchi et al. 2023 (three individuals) and *Acutuncus mecnuffi* Vecchi et al. 2023 (two individuals) were obtained from rock pool sediments collected during a previous study (Vecchi et al. 2026). Sample information is provided in Table 1. Tardigrades were isolated and manipulated with flame‐sterilised tungsten Irwin loops and kept overnight in sterile distilled water at 8°C to empty their gut contents.

**TABLE 1:** Information overview of the sequenced individuals and sequencing run statistics, divided by individual and statistics for reads identified, as of putative mitochondrial origin by DIAMOND blastp.

Sample	Species	Country	Coordinates	Sum Gb raw reads [N50 bp—average quality Q]	Sum Gb mitochondrial reads [N50 bp—average quality Q]	% mitochondrial reads
Acu_gio_RIT094	*Acutuncus giovanniniae*	Italy	44.381019, 10.047973	18.11 [5509–19.20]	0.16 [8126–17.80]	0.73
Acu_gio_RPL027	*Acutuncus giovanniniae*	Poland	50.777043, 15.590996	16.99 [6637–19.46]	2.34 [5850–19.68]	15.78
Acu_gio_RSE010	*Acutuncus giovanniniae*	Sweden	56.138949, 14.831353	13.18 [5041–19.37]	6.01 [4370–19.08]	52.26
Acu_mec_RSE086	*Acutuncus mecnuffi*	Sweden	56.155758, 14.876410	13.02 [7123–19.39]	0.51 [6998–19.00]	4.81
Acu_mec_RSE087	*Acutuncus mecnuffi*	Sweden	56.156057, 14.877798	12.70 [7612–19.63]	0.16 [8997–18.53]	1.38

### 2.2 Whole Genome Amplification and Sequencing

Tardigrades were transferred with a sterilised tungsten Irwin loop into 4 μL of REPLI‐g Advanced Storage buffer on a sterilised glass slide and cut with an insulin needle to release the body cavity content, and then, the buffer containing the cells was transferred to a microcentrifuge vial. Whole genome amplification was then done following the REPLI‐g Advanced DNA Single Cell Kit manufacturer's protocol. The reaction product was purified with a GeneMAGNET PCR/DNA Clean‐Up Purification Kit (EURx), and the ds‐DNA was quantified using a Qubit Fluorometric assay. Approximately 2 μg of amplified DNA was debranched for 1 h with T7 Endonuclease I (New England Biolabs) according to the manufacturer's protocol. The reaction product was again purified with GeneMAGNET and quantified with Qubit. About 1000 ng of the amplified DNA for each sample was used to prepare a library according to the Native Barcoding Kit 24 V14 SQKNBD114.24 protocol (Oxford Nanopore Technologies Limited, Oxford, UK). All samples were processed fully independently until after the addition of barcodes. The samples were combined into two pools, loaded on R10.4.1 PromethION flow cells (FLO‐PRO114) and sequenced on a PromethION 24 instrument (MinKnow 24.06.15). Basecalling was performed using the dorado basecaller server 7.4.14 set to ‘super‐accurate’ mode, 400 bp, 5 Hz. Reads with *Q* < 10 were discarded. Library preparation, sequencing and demultiplexing were performed by a commercial provider (IGA Technology, Udine, Italy). Residual adapters were removed with Porechop_ABI (Bonenfant et al. 2023).

### 2.3 Assembly

To speed up the assembly process, mitochondrial reads were first extracted as in Vecchi and Stec (2025): in brief, the reads were blasted with DIAMOND blastx (Buchfink et al. 2021) against a database of tardigrade mitochondrial proteins (see Vecchi and Stec 2025) and reads with at least one match were retained. Two assembly approaches were then tested: one with long reads (hereafter referred to as LR assembly), and one based on fragmentation of long reads into short reads (hereafter referred to as SR assembly).

### 2.4 LR Assemblies

Multiple assemblies per sample were produced with the software Flye v 2.9.3 (Kolmogorov et al. 2020; Lin et al. 2016). The first assembly was produced with the default Flye mode with 1 polishing iteration. The second was produced with the Flye—meta option with 1 polishing iteration, and the third was produced with the Flye—gsize option (genome size specified to 14.5 kb based on preliminary assembly tests) with 1 polishing iteration. The assembly process, as explained, was then repeated for the unmodified reads and for the reads with palindromes removed with the script provided by Lee et al. (2023), with two different minimum alignment lengths to classify a read as palindromic of 100 and 1000 bp. The second length of 1000 bp is the default value proposed in the original script of Lee et al. (2023), while 100 bp was chosen to provide a much more extreme palindrome detection. The process generated a total of nine assemblies for each sample in a crossed design: assembly algorithm (Flye standard, Flye meta, Flye gsize) * palindrome treatment (no treatment, alignment length 1000, alignment length 100). Assembly success was evaluated by the presence of circular contigs with a size close to 14.5 kb. The identity of the contigs (i.e., the contig is a mitochondrial genome and belongs to the right species) was then confirmed by blasting the sequence against the NCBI core_nt database (Sayers et al. 2025) with blastn (Camacho et al. 2009) using default parameters.

### 2.5 SR Assembly

To use an assembler designed for short‐read sequencing technology (i.e., Illumina) before assembly, the reads were chopped into 150 bp fragments, and only fragments *Q* ≥ 30 were retained using seqkit v 2.10.1 (Shen et al. 2016). Then, the mitogenomes were assembled with NOVOPlasty v.4.3.5 with k‐mer size 33 (Dierckxsens et al. 2016) using COI sequences from either *A. giovanniniae* or *A. mecnuffi* from GenBank (MW306855 Vecchi et al. 2022; OM350039 Vecchi et al. 2023). Assembly success was evaluated as above.

### 2.6 Mitogenomes Annotation

The mitochondrial genomes were annotated with MITOS2 (Bernt et al. 2013; Donath et al. 2019). The annotation was inspected and curated by hand, and a GenBank flat file was produced using the scripts mitos2fasta.py and aln2tbl.py (https://github.com/IMEDEA/mitogenomics) and table2asn (https://ftp.ncbi.nlm.nih.gov/asn1‐converters/by_program/table2asn/). The mitogenomes were visualised with OGDRAW (Greiner et al. 2019).

### 2.7 Palindromes Quantification in Reads

Palindromes were quantified by blasting the long reads against the respective mitogenome assembly with blastn (Camacho et al. 2009; E‐value treshold 1e−5, identity threshold 95%). A read was considered palindromic if multiple mitogenome matches were present in different orientations. Processing of blastn results and results visualisation were performed with ‘R v 4.5.2’ (R Core Team 2022) using the packages ‘tidyverse v 2.0.0’ (Wickham et al. 2019), ‘ggplot2 v 4.0.2’ (Wickham 2016), ‘ggridges v 0.5.7’ and ‘gggenes v 0.6.0’.

### 2.8 Code Availability

All the code used for this study is available at https://doi.org/10.5281/zenodo.19131525 and as Supporting Information SM.01.

## 3 Results

The sequencing run produced about 12–18 Gb of sequences with average quality above Q19 (see Table 2). Read length N50 was between 3743 and 5304 bp, which can be considered low for standard Nanopore whole genome sequencing runs. This was expected as the fragmentation of the DNA library was worsened by the WGA and debranching process. The raw reads were submitted to NCBI SRA (Bioproject PRJNA1287536).

**TABLE 2:** Mitogenome assembly statistics for the different methods expressed as assembly size in bp [coverage].

	Flye (LR)									NOVOplasty
	Raw reads			Palindrome 1000			Palindrome 100			
	Flye	Flye meta	Flye gsize	Flye	Flye meta	Flye gsize	Flye	Flye meta	Flye gsize	
Acu_gio_RSE010	n.o.	14,913 [38]	n.o.	n.o.	14,398 [63]	n.o.	n.o.	n.o.	n.o.	14,416 [343,268]
Acu_gio_RPL027	n.o.	n.o.	n.o.	n.o.	n.o.	n.o.	n.o.	n.o.	n.o.	14,417 [128,066]
Acu_gio_RIT094	n.o.	n.o.	n.o.	14,469 [663]	14,462 [682]	n.o.	14,470 [810]	14,440 [635]	n.o.	14,415 [4113]
Acu_mec_RSE086	n.o.	n.o.	n.o.	n.o.	n.o.	n.o.	n.o.	n.o.	n.o.	14,398 [22,115]
Acu_mec_RSE087	n.o.	n.o.	n.o.	n.o.	n.o.	n.o.	n.o.	n.o.	n.o.	14,399 [4325]

Abbreviation: *n.o*., indicates that a circular mitogenome of the approximately right size and of the right species was not obtained.

The number of putative mitochondrial reads identified by blastx against a tardigrade mitochondrial protein database varied greatly across samples (Table 1), from 0.73% to 52.23% of all reads. The proportion of mitochondrial reads measured as the number of reads with a blast match against the final mitogenome assembly (see paragraphs below) produced similar numbers identified with the method mentioned above (Table 1).

### 3.1 LR Assembly

Circularised assemblies were produced by *Flye* only for two individuals (Table 2) belonging to *A. giovanniniae*. Across the same individual, non‐negligible differences in assembly size are found (ranging from 14,398 to 14,913 bp in *A. giovanniniae*, surpassing any plausible intraspecific variability). Flye in genome size mode never produced an assembly, while Flye in metagenome mode performed the best. Due to the low success rate, heterogeneity in assembly lengths and relatively low coverage compared to the SR assemblies (see below), the obtained LR assemblies were considered unsuccessful and not used for other downstream analyses.

### 3.2 SR Assembly and Mitogenome Annotation

**FIGURE 1:**
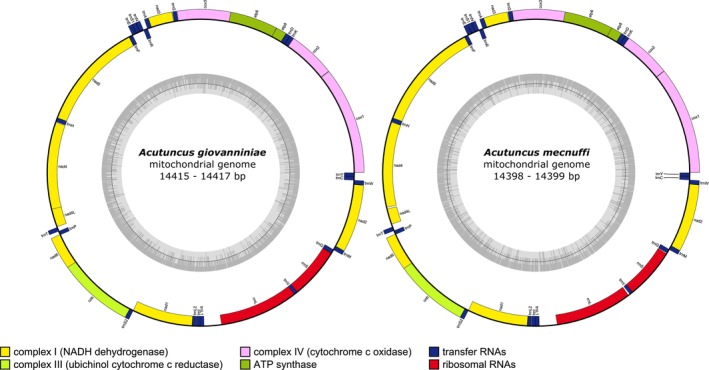
Graphical representation of mitochondrial genomes of *A. giovanniniae* and *A. mecnuffi*.

The short‐reads assembly method produced a circularised assembly of the expected size for each individual (Table 2), with negligible length differences between individuals of the same species (3 bp for *A. giovanniniae* and 1 bp for *A. mecnuffi*). The automatic annotation with MITOS2 of the mitogenomes obtained with the SR assembly produced almost complete annotations (13 protein coding genes, 22 tRNAs, two ribosomal RNAs; Figure 1), with the exceptions of the absence of ATP synthase subunit 8 (atp8) in *A. giovanniniae* (which was found to be present by a manual curation) and the false positive presence of two copies of NADH dehydrogenase subunit 4 L (nad4L) in *A. mecnuffi*, which was also revealed to be an error solved after manual curation. Because no reference mitogenomes from the same genus were available for comparison, the accuracy of the SR assemblies was evaluated indirectly. Their complete circularisation, negligible intraspecific length variation and successful annotation of all genes usually present in tardigrade mitogenomes supported their interpretation as the most accurate assemblies. The five mitogenomes are available on GenBank under accession numbers PZ148665‐9.

### 3.3 Palindromes Quantification in Reads

**FIGURE 2:**
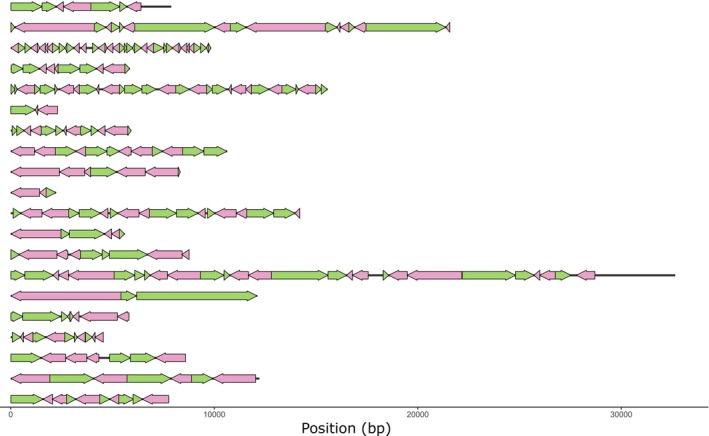
Example of palindromic reads (random sample). Arrows indicate blastn matches to reference mitogenomes according to match orientation. Colour highlights alternative matching directions.

Palindrome quantification was performed by aligning the long reads used for the LR assembly (i.e., only reads matching the mitochondrial proteins database) against their respective mitogenome obtained through SR assembly. Reads with multiple matches in different orientations to their respective genomes were considered as palindromic. The majority of the reads had a blastn match to their mitogenome (Table 3), and about half of the reads selected for the assembly by blastx against a mitochondrial database, which matched the final mitogenome assemblies (48.39%–56.00%; Table 3), had only one match. Among the reads with at least two matches to the mitogenome, more than two‐thirds (68.41%–74.51%; Table 3) were identified as palindromic. Examples of the structure of those palindromic reads are presented in Figure 2.

**TABLE 3:** Results of palindrome quantification in reads through blastn against the corresponding assembled mitogenome.

Individual	# of reads	# of reads matched to mitogenome	# of reads with one match to the mitogenome	# of palindromic reads	% reads with one match to the mitogenome	% of palindromic reads (over all matched reads)	% of palindromic reads (over reads with at least two matches to the mitogenome)
Acu_gio_RIT094	30,888	27,103	13,114	10,423	48.39	38.46	74.51
Acu_gio_RPL027	535,322	522,167	264,184	178,060	50.59	34.1	69.02
Acu_gio_RSE010	1,840,681	1,810,510	859,350	653,968	47.46	36.12	68.75
Acu_mes_RSE086	125,643	109,818	61,499	33,365	56.00	30.38	69.05
Acu_mes_RSE087	32,919	22,398	14,028	5726	62.63	25.56	68.41

## 4 Discussion

The introduction of WGA has been a key technological innovation, enabling the genomic characterisation of microscopic taxa for which the limiting factor for a long time was low DNA content. We documented and evaluated how the use of MDA, which, due to its high reaction speed and amplification gain, introduces palindromic artefacts into mitochondrial genomes, affects mitogenome assembly after long‐read sequencing and how this issue can be mitigated. We demonstrated that, while long‐read assemblies based on MDA frequently fail in the presence of pervasive palindromes, simple in silico fragmentation of long reads into short, high‐quality fragments enhances robust and reproducible mitogenome reconstruction.

The application of MDA to single microscopic creatures has facilitated their genome and mitogenome sequencing, but the formation of palindromic reads still represents an issue. In our study system, roughly one‐third of all mitochondrial reads were palindromic (Table 3), that is, matches to the mitochondrial genomes in different directions were present. This proportion of palindromic reads is comparable with other similar studies (Lu et al. 2023), but the complex structure of palindromic repeats found in this study (Figure 2) was not reported before. Palindromic read generation can potentially be explained by the high reaction gain of the REPLI‐g Advanced Single Cell kit used for this study (which can yield up to 35 μg of the amplified product from a single cell). It could be hypothesised that the high reaction gains and reaction speed (2 h) could increase the probability of multiple primers annealing with the template in the same region, with consequent strand displacement and palindrome formation. To support this association between reaction speed and palindrome formation, two previous studies (Camarda et al. 2025; Vecchi and Stec 2025) did not find any assembly issues on tardigrade mitogenomes while using an almost identical protocol to this study. The main difference was the use of a REPLI‐g Mini Kit (Qiagen), which has lower yield and a much longer reaction time (10–16 h vs. 2 h in the REPLI‐g Advanced Single Cell kit). Similar amplification‐associated palindrome formation has been documented in long‐read datasets derived from single nematodes (Lee et al. 2023) and other microscopic organisms (Agyabeng‐Dadzie et al. 2025), but it has never been precisely quantified. Our findings therefore provide empirical evidence that palindromic read formation is not a marginal artefact but can be a dominant feature of high‐gain and high‐speed MDA in mitogenomes.

### 4.1 Long‐Read Assembly in the Presence of Pervasive Palindromes

Assembly attempts directly on reads with a high proportion of palindromes have proven mostly unsuccessful (Table 3). Long‐read assemblers such as Flye rely on repeat graph construction and assume that read structure reflects contiguous genomic segments (Lin et al. 2016). Extensive palindromic inversions violate this assumption, artificially introducing inverted repeats and structural complexity that can fragment assembly graphs or promote misassembly. Bioinformatic tools to correct MDA‐induced palindromes in long reads have already been devised, and after identifying palindromic reads, they either discard them (Agyabeng‐Dadzie et al. 2025) or split them at detected inverted alignments (Lee et al. 2023; Lu et al. 2023; Warris et al. 2018). While the first approach can result in a non‐negligible data loss when palindromic reads are present at high abundance, the second approach mostly works when palindromic repeats are long (above a user‐specified threshold) and only one or two inversion events are present in each palindromic read. In our data, most of the palindromic reads contain complex multi‐segment structures (Figure 2), suggesting repeated strand‐displacement and re‐priming events and effectively preventing mitogenome assembly. Moreover, the two mentioned correction approaches rely primarily on aligning the reads to their reverse complement and flagging them as palindromic if matches above a certain threshold are found. However, these methods are computationally intensive as they require an additional mapping or alignment step.

### 4.2 Read Fragmentation as a Robust Solution to Mitogenome Assembly

While palindrome detection and correction have been shown to be effective for nuclear genomes, a different strategy proved more suitable for mitochondrial genomes. In silico fragmentation of long reads into 150 bp high‐quality fragments, followed by short‐read assembly with NOVOPlasty, consistently yielded circular mitogenomes of the expected size with negligible and biologically plausible length variation across individuals. Importantly, coverage values in the short‐read assemblies were several orders of magnitude higher than those obtained with long‐read assemblies. While in our case, due to the high sequencing depth, coverage was not an issue, in some cases, when economical constraints limit the number of obtainable reads, the higher efficiency of the proposed methods provides clear advantages. The fragmentation eliminates the structural artefacts by breaking the long palindromic sequences into segments that represent the true mitochondrial sequences, but without the problematic and erroneous structural information. Only a small number of short fragments will retain the junctions between inverted repeats; the successful assemblies show that those still problematic reads are not numerous enough to cause issues for assemblers. While long‐range spatial information is lost, it is inconsequential when dealing with small circular genomes (~14.5 kb) that can easily be assembled from short overlapping fragments. As mitochondrial genomes are circular, small and in many cases lack extensive repeats, they are especially amenable to this strategy. For nuclear genome assembly, however, fragmentation would sacrifice essential long‐range information and would not constitute a viable solution. Overall, the proposed read‐fragmentation approach provides a practical and robust alternative to previously existing tools for palindrome removal (Lee et al. 2023; Lu et al. 2023; Warris et al. 2018) for the assembly of small genomes that naturally lack extensive repeated sequences, where there is less need for the long‐range spatial information provided by long reads and in which MDA has produced complex palindromic structures.

### 4.3 Applications, Limitations and Future Directions

From the results presented here, it would seem advantageous to sequence MDA amplified libraries with short‐read technologies instead of long‐reads (like Nanopore), as long reads would need to be fragmented anyway before mitogenome assembly. However, Nanopore sequencing platforms offer practical advantages over commercial short‐read platforms: devices are relatively inexpensive and scalable, allowing sequencing to be performed in small laboratories or even field environments, and sequencing runs can be monitored in real time to stop once sufficient coverage has been obtained (Menegon et al. 2017; Parker et al. 2017). Information provided by long reads could still be used for the assembly of nuclear genomes, for which already available palindrome‐correction tools have been shown to be effective (Lee et al. 2023). Even when short‐read assembly strategies are used, generating data through Nanopore sequencing remains a powerful and versatile approach.

An important limitation of this approach lies in the inherently high indel error rate of Nanopore sequencing compared to Illumina (Chen et al. 2025). In our approach, the fragmented Nanopore reads are selected for quality *Q* > 30; however, the error profile could still be different than true Illumina reads, in particular regarding indels. This could lead to errors in the mitogenome assembly by software designed specifically for Illumina sequences, and therefore, assembled mitogenomes must be carefully inspected, and particular care should be given to check for the presence of complete ORFs, which would be easily disrupted by indels introduced in the assembly process.

While the current study used tardigrades as a model to develop the proposed mitogenome assembly pipeline, the approach is extremely flexible regarding taxonomy: any dataset derived from long‐read sequencing of MDA amplified genomes has the potential to result in the formation of palindromic reads, which could then benefit from the approach proposed here when assembling the mitogenome. The main purpose of read pre‐filtering based on a mitochondrial protein database in this study was to reduce the number of reads to process; for sequencing runs with lower outputs, this step may not be needed at all. For sequencing runs with lower output, this step may not be needed. If pre‐filtering is required, the taxonomic limitation imposed by a reference database can be reduced by using a custom database generated for the organism or group of interest.

Also, the precise detection and quantification of palindromic reads require mapping them to a reference genome (Lu et al. 2023), which in our case was easy to obtain, but which is not always the case with the bigger and more complex nuclear genomes. Published research shows that in nematodes the presence of palindromes in nuclear reads can be easily solved with the palindromic reads' detection and correction bioinformatic tools mentioned above (Lee et al. 2023). However, in this study, the authors used an MDA kit with lower gain and reaction speed; thus, the outcome cannot be easily compared with our results. Future research should try to quantify the influence of diverse MDA kits and genomic DNA input characteristics on the production of palindromic sequences.

To summarise, this study showed that high reaction‐gain and high‐speed MDA applied to low‐biomass specimens can generate pervasive palindromic reads that severely impair long‐read mitogenome assembly. However, simple in silico fragmentation of long reads into artificial short reads enables consistent and accurate reconstruction of circular mitochondrial genomes. This strategy provides a practical and scalable solution for mitogenomics of microscopic animals and supports broader efforts to reduce taxonomic bias in genomic resources.

## Author Contributions

**Matteo Vecchi:** conceptualisation, data curation, formal analysis, funding acquisition, investigation, methodology, project administration, resources, supervision, validation, visualisation, writing – original draft, writing – review and editing. **Bartłomiej Surmacz:** validation, writing – review and editing. **Ingemar K. Jönsson:** resources, writing – review and editing. **Daniel Stec:** conceptualisation, project administration, supervision, writing – review and editing.

## Funding

This study was funded by the Polonez Bis grant No. 2022/45/P/NZ8/01512 to M.V. co‐funded by the National Science Centre and the European Union framework Programme for Research and Innovation Horizon 2020 under the Marie Skłodowska‐Curie grant agreement [No. 945339].

## Ethics Statement

The authors have nothing to report.

## Consent

The authors have nothing to report.

## Conflicts of Interest

The authors declare no conflicts of interest.

## Supporting information


**Supporting Information: S1.** Code used in the manuscript analyses.

## Data Availability

All the data and code produced in this study are available as Supporting Information of this article (Code used for the analyses: SM.01) and/or deposited in open‐access databases (Raw reads: NCBI SRA BioProject PRJNA1287536; Assembled mitogenomes: GenBank PZ148665‐9; Code used for the analyses: https://doi.org/10.5281/zenodo.19131525 and SM.01).
